# Single Nucleotide Polymorphisms of the Angiotensin-Converting Enzyme (*ACE)* Gene Are Associated with Essential Hypertension and Increased ACE Enzyme Levels in Mexican Individuals

**DOI:** 10.1371/journal.pone.0065700

**Published:** 2013-05-31

**Authors:** Nancy Martínez-Rodríguez, Carlos Posadas-Romero, Teresa Villarreal-Molina, Maite Vallejo, Leonardo Del-Valle-Mondragón, Julian Ramírez-Bello, Adan Valladares, Miguel Cruz-López, Gilberto Vargas-Alarcón

**Affiliations:** 1 Molecular Biology Department, Instituto Nacional de Cardiología Ignacio Chávez (INCICH), Mexico City, Mexico; 2 Endocrinology Department, INCICH, Mexico City, Mexico; 3 Genomic Laboratory of Cardiovascular Diseases, Instituto Nacional de Medicina Genómica (INMEGEN), Mexico City, Mexico; 4 Sociomedical Department, INCICH, Mexico City, Mexico; 5 Pharmacology Department, INCICH, Mexico City, Mexico; 6 Immunogenetics Laboratory, INMEGEN, Mexico City, Mexico; 7 Biochemistry Unit, Centro Médico Siglo XXI, Mexico City, Mexico; 8 Graduate Studies in Biomedical Sciences, Universidad Nacional Autónoma de México, Mexico City, Mexico; Max-Delbrück Center for Molecular Medicine (MDC), Germany

## Abstract

**Aim:**

To explore the role of the *ACE* gene polymorphisms in the risk of essential hypertension in Mexican Mestizo individuals and evaluate the correlation between these polymorphisms and the serum ACE levels.

**Methods:**

Nine *ACE* gene polymorphisms were genotyped by 5′ exonuclease TaqMan genotyping assays and polymerase chain reaction (PCR) in 239 hypertensive and 371 non- hypertensive Mexican individuals. Haplotypes were constructed after linkage disequilibrium analysis. *ACE* serum levels were determined in selected individuals according to different haplotypes.

**Results:**

Under a dominant model, rs4291 rs4335, rs4344, rs4353, rs4362, and rs4363 polymorphisms were associated with an increased risk of hypertension after adjusting for age, gender, BMI, triglycerides, alcohol consumption, and smoking. Five polymorphisms (rs4335, rs4344, rs4353, rs4362 and rs4363) were in strong linkage disequilibrium and were included in four haplotypes: H1 (*AAGCA*), H2 (*GGATG*), H3 (*AGATG*), and H4 (*AGACA*). Haplotype H1 was associated with decreased risk of hypertension, while haplotype H2 was associated with an increased risk of hypertension (OR = 0.77, *P* = 0.023 and OR = 1.41, *P* = 0.004 respectively). According to the codominant model, the H2/H2 and H1/H2 haplotype combinations were significantly associated with risk of hypertension after adjusted by age, gender, BMI, triglycerides, alcohol consumption, and smoking (OR = 2.0; *P* = 0.002 and OR = 2.09; *P* = 0.011, respectively). Significant elevations in serum ACE concentrations were found in individuals with the H2 haplotype (H2/H2 and H2/H1) as compared to H1/H1 individuals (*P* = 0.0048).

**Conclusion:**

The results suggest that single nucleotide polymorphisms and the “*GGATG*” haplotype of the *ACE* gene are associated with the development of hypertension and with increased ACE enzyme levels.

## Introduction

Hypertension plays a major etiologic role in the development of cerebrovascular disease, ischemic heart disease, cardiac and renal failure. [1]. Essential hypertension is a complex disease where environmental, demographic, and genetic factors are involved [2], [3]: It has been estimated that approximately 30% of the interindividual variability in blood pressure is genetically determined [4]. Special attention has been given to the role of genetic variation in genes implicated in the renin-angiotensin system (RAS), particularly the angiotensin-converting enzyme (*ACE*) gene [5], [6]. ACE is a key enzyme in the renin-angiotensin-aldosterone (RAAS) and kalikrein-kinin systems [7], [8] playing a crucial role in blood pressure (BP) regulation and electrolyte balance [9]. The most studied polymorphism in the RAAS system is an insertion or deletion (I/D) of a 287 bp sequence of DNA in intron 16 of the *ACE* gene. It has been suggested that this polymorphism could explain up to 47% of total phenotypic variation in ACE serum levels and determines ACE enzyme activity [10]. ACE serum levels of *D/D* homozygous individuals are reported to be twice as high as those of *I/I* homozygous individuals, while *I/D* heterozygous individuals have intermediate ACE levels [11]. It is currently believed that the *I/D* polymorphism is not directly responsible for inherited ACE serum level variation in humans [10], [12], [13]. Tiret et al. demonstrated that this polymorphism is in close linkage disequilibrium to at least one, and perhaps more functional polymorphisms determining the phenotypic variations of enzyme levels [11]. Still, other studies have shown loci with variants of this sequence in complete linkage disequilibrium with the *I/D* polymorphism [14]. The role of *ACE* gene polymorphisms in hypertension has not been studied in the Mexican population. We selected 9 polymorphisms considering a previous study in which *ACE* haplotypes were described in African-American and European-American populations [15]. Population LD block differences were observed, as haplotypes were shorter and more diverse in Africans, while Europeans had longer and fewer haplotypes. This suggests that different linkage disequilibrium of the polymorphisms located in the *ACE* gene can be observed in other populations. On the other hand, the length of the genomic segment covered by the polymorphisms included in our study is 21 kb, increasing the possibility to detect some association with the disease. Thus, the objective of the present study was to analyze whether these polymorphisms or given haplotypes are associated with essential hypertension and ACE serum levels in Mexican Mestizo individuals.

## Materials and Methods

### Subjects

All participants provided written informed consent, and the study complies with the Declaration of Helsinki and was approved by the Ethics Committee of the Instituto Nacional de Cardiología ‘‘Ignacio Cháávez’’ (INCICH)**.** This study included a group of 239 hypertensive individuals (120 men and 119 women) referred to the National Institute of Cardiology (INCICH) in Mexico City. Hypertension was defined as systolic blood pressure (BP) ≥140 mmHg, diastolic BP ≥90 mmHg, or the use of at least one class of antihypertensive drugs. One hundred hypertensive subjects (41.8%) taking antihypertensive drugs (64 patients one drug, 26 patients two drugs and 10 patients three drugs). The drugs used were ACE inhibitors in 37 patients, beta-blockers in 38, angiotensin II type-1 receptor antagonist in 33, diuretics in 28 and calcium channel antagonists in 18. Secondary hypertension was minimized using detailed health questionnaire and clinical evaluation, and none patient had evidence of cardiac or renal failure. A group of 371 non-hypertensive individuals (168 men and 203 women) were recruited among blood donors at the INCICH with systolic and diastolic blood pressures below 140 and 90 mmHg respectively. All participants were ethnically matched, defining Mexican Mestizos as individuals born in Mexico for three generations, including their own. All participants provided informed consent.

### Genetic analysis

Genomic DNA from whole blood containing EDTA was isolated by standard techniques [16]. The *A-239T* (rs4291), *A7941G* (rs4318), *A10539G* (rs4335), *A11599G* (rs4343), *A12292G* (rs4344), *A15990G* (rs4353), *C19329T* (rs4362), and *A20069G* (rs4363) single nucleotide polymorphisms (SNPs) were genotyped using 5’ exonuclease TaqMan genotyping assays on an ABI Prism 7900HT Fast Real-Time PCR system, according to manufacturer’s instructions (Applied Biosystems, Foster City, CA, USA) (Table S1). The *ACE* polymorphism on intron 16 (I/D) was determined by PCR using the following primers: sense 5′-CTGCAGACCACTCCCATCCTTTCT-3′ and antisense 5′-GATGTGGCCATCACATTCGTCAGAT-3′ previously described [10]. Each sample found to have the *D/D* genotype was subjected to a second PCR amplification with insertion-specific primers (5a: 5′-TGGGACCACAGCGCCCGCCACTAC-3′ and 5c: 5′-TCGCCAGCCCTCCCATGCCCATAA-3′) in order to avoid *D/D* mistyping [17].

Pairwise linkage disequilibrium (LD, D) estimations between polymorphisms and haplotype reconstruction were performed with Haploview version 4:1 (Broad Institute of Massachusetts Institute of Technology and Harvard University, Cambridge, MA, USA).

### Serum ACE levels

Serum ACE levels were measured in 169 hypertensive individuals selected according to haplotype (33 H1/H1, 94 H1/H2 heterozygous and 42 H2/H2 homozygous). ACE concentrations were determined with Boster’s human ACE ELISA Kit that was based on standard sandwich enzyme-linked immune-sorbent assay technology; measurements were in picograms per milliliter (pg/ml.).

### Statistical analysis

Demographic and clinical variables between hypertensive and non-hypertensive groups were analyzed with Stata 8.0 for Windows software. Numerical variables not normally distributed are presented as median and percentiles 25 and 75, and were compared using Mann Whitney U test. Hardy-Weinberg equilibrium (HWE) and comparisons between categorical variables were analyzed using Chi^2^. Logistic regression analyses were used to test for associations of polymorphisms or haplotypes with hypertension under four inheritance models: co-dominant, dominant, recessive, and heterozygous advantage. The most appropriate inheritance model was selected based on Akaike information criteria (AIC) and was adjusted by age, gender, BMI, triglycerides, alcohol consumption, and smoking. Statistical power to detect association of polymorphisms with hypertension exceeded 0.80 as estimated with QUANTO software (http://hydra.usc.edu/GxE/).

Correlation between serum ACE concentration and haplotypes was tested using the Mann-Whitney U test. Statistical significance was set at *P*≤0.05.

### Functional prediction analysis

We predicted the potential effect of the *ACE* polymorphisms associated with hypertension in our population using bioinformatics Tools, including FastSNP [18], SNP Function Prediction (http://snpinfo.niehs.nih.gov/snpfunc.htm), Human-transcriptome DataBase for Alternative Splicing (http://www.h-invitational.jp/h-dbas/), SplicePort (http://www.spliceport.cs.umd.edu/SplicingAnalyser2.html), ESE finder (http://rulai.cshl.edu/cgi-bin/tools/ESE3/esefinder.cgi), HSF (http://www.umd.be/HSF/) and SNPs3D (http://www.snps3d.org/).

## Results

### Characteristic of the study sample

Demographic and clinical characteristics of patients and controls are shown in Table 1. Body mass index, triglyceride levels, glucose levels and alcohol consumption were significantly higher in hypertensive than in non-hypertensive individuals; while mean age, sex ratio and cholesterol levels were similar in both groups.

**Table 1:** Comparison of cardiovascular risk factors in hypertensive and non-hypertensive individuals.

		Hypertensive (n = 239)			Non-hypertensive (n = 371)			*P value*
		P25	median	P75	P25	median	P75	
Age (years)		51	58	63	52	56	62	0.066
Body-mass Index (kg/m2)		26.8	29.3	32.1	24.5	27.1	29.8	**<10^−3^**
Triglycerides (mg/dl)		117	160	215	105	142	191	**0.003**
Glucose (mg/dl)		88	95	108	83	90	115	**<10^−3^**
Cholesterol (mg/dl)		175.3	196	215	166	193	211	0.083
HDL(mg/dl)		36	44	54	37	45	56	0.161
LDL(mg/dl)		99.6	121.4	138	98	118.8	137	0.319
Blood Pressure (mmHg)	Systolic	127	137.5	151	103	111.5	121	**<10^−3^**
	Diastolic	74.3	81.5	87.5	65	69.5	76	**<10^−3^**
Gender (n(%))	Male	120(50.2)			168(45.3)			0.134
	Female	119(49.8)			203(54.7)			
Tobacco smoking (n(%))	Yes	43(18.0)			104(28.0)			**0.003**
Use of alcohol (n(%))	Never used	94 (39)			177 (48)			**0.025**
	> = 6 g/day use	145 (61)			194 (52)			
Type 2 diabetes mellitus (n(%))	Yes	33 (14)			71 (19)			0.054
Family history of hypertension (n(%))	Yes	121 (51.0)			231 (62.0)			**0.005**

Data are expressed as median and percentiles 25 and 75.

* P values were estimated using Mann-Whitney U-test continuous variables and Chi-square or Fisher test for categorical values.

### Allele and genotype frequencies

No deviation from HWE was observed for rs4291, rs4318, rs4335, rs4344, rs4353, rs4362, and rs4363 polymorphisms; however, the rs4343 was not in HWE in the non-hypertensive individuals. Allele frequencies of rs4291, rs4335, rs4344, rs4353, rs4362 and rs4363 differed significantly between hypertensive and non-hypertensive individuals. According to the dominant model, rs4291 (OR = 1.5, 95%CI: 1.07–2.15, *P* = 0.02,), rs4335 (OR = 2.05, 95%CI: 1.39–2.99, *P*<10^−3^), rs4344 (OR = 1.82, 95%CI: 1.24–2.68, *P* = 0.002), rs4353 (OR = 1.80, 95%CI: 1.22–2.64, *P* = 0.003), rs4362 (OR = 1.71, 95%CI: 1.17–2.51, *P* = 0.006), and rs4363 (OR = 1.87, 95%CI: 1.27–2.74, *P* = 0.001,) were significantly associated with hypertension adjusting for age, gender, BMI, triglycerides, alcohol consumption, and smoking (Table 2).

**Table 2:** Associations of *ACE* polymorphisms with hypertension.

Polymorphism	Alleles*	MAF^a^	MAF^a^	Genotypes	Genotypes	OR(95%CI);
		HT	NHT	HT	NHT	P*_dom_* value
*A-239T* (rs4291)	*T/A*	0.35	0.29	25/118/96	34/149/188	1.51 (1.07–2.15); 0.020
				0.11/0.49/0.40	0.09/0.40/0.51	
*A7941G* (rs4318)	*G/A*	0.01	0.02	0/7/232	0/12/359	NS
				0.0/0.03/0.97	0.0/0.03/0.97	
*A10539G* (rs4335)	*G/A*	0.47	0.38	47/130/62	55/169/147	2.05 (1.39–2.99); **<**10^−3^
				0.20/0.54/0.26	0.15/0.45/0.40	
*I/D* (rs4646994)	*I/D*	0.22	0.25	6/95/138	18/147/206	NS
				0.02/0.40/0.58	0.05/0.40/0.55	
*A11599G* (rs4343)	*G/A*	0.42	0.37	41/117/81	39/196/136	NS
				0.17/0.49/0.34	0.10/0.53/0.37	
*A12292G* (rs4344)	*G/A*	0.49	0.42	55/124/60	69/171/131	1.82 (1.24–2.68); 0.002
				0.23/0.52/0.25	0.19/0.46/0.35	
*A159990G* (rs4353)	*A/G*	0.48	0.41	49/129/61	67/171/133	1.80 (1.22–2.64); 0.003
				0.20/0.54/0.26	0.18/0.46/0.36	
*C19329T* (rs4362)	*T/C*	0.46	0.40	44/133/62	65/170/136	1.71 (1.17–2.51); 0.006
				0.18/0.56/0.26	0.17/0.46/0.37	
*A20060G* (rs4363)	*G/A*	0.47	0.40	47/129/63	62/170/139	1.87 (1.27–2.74); 0.001
				0.20/0.54/0.26	0.17/0.46/0.37	

Adjusted for age, gender, BMI, triglycerides, alcohol consumption and smoking.

a : MAF, minor allele frequency.

HT: Hypertensive individuals.

NHT: Non-hypertensive individuals.

NS: Not significant.

* Underlined letter denotes the minor allele in the control samples.

### SNP function prediction

Based on SNP functional prediction software’s, only the rs4335 (FastSNP software), rs4353 (FastSNP software), and rs4363 (ESE finder and HSF software’s) polymorphisms seem to be functional. The variation in these polymorphisms affects the DNA binding of the transcription factors AML-1, GATA-1, and Srp40, respectively.

### Haplotype frequencies

**Figure 1:**
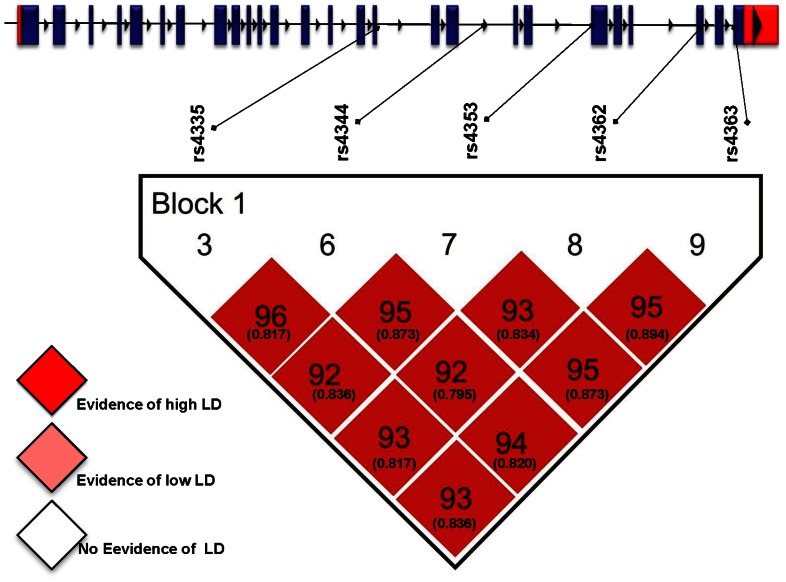
Haploview linkage disequilibrium graph of five *ACE* gene polymorphisms (rs4335, rs4344, rs4353, rs4362, rs4363). Pairwise linkage disequilibrium coefficients D ´× 100 are shown in each cell (D ´ values of 1.0 are not shown). Standard color scheme of Haploview was applied for linkage disequilibrium color display (logarithm of odds [LOD] score ≥2 and D ´  = 1, shown in bright red; LOD score ≥2 and D ´**<**1 shown in shades of pink/red; LOD score ≤2 and D ´<1 shown in white). D values of 1.0 are not shown. Numbers in boxes and parentheses represent D values and r2 values after the decimal point, respectively.

The linkage disequilibrium analysis was made considering the nine studied polymorphisms (Fig S1). Five polymorphism (rs4335, rs4344, rs4353, rs4362, rs4363) with high linkage disequilibrium (D’ values >0.9 and r2 values >0.8) were reanalyzed (Fig 1) and used to construct four haplotypes, H1 (*AAGCA*), H2 (*GGATG*), H3 (*AGATG*), and H4 (*AGACA*). The H1 haplotype was associated with decreased risk (OR = 0.77, 95%CI: 0.66–0.97, *P* = 0.023), while the H2 haplotype was associated with increased risk of hypertension (OR = 1.41, 95%CI: 1.11–1.80, *P* = 0.004) (Table 3). Afterwards, the most common haplotypes were tested for association with hypertension under different inheritance models. Under the codominant model H2/H2 and H1/H2 haplotype combinations were significantly associated with risk of hypertension after adjusted by age, gender, BMI, triglycerides, alcohol consumption, and smoking (Table 4).

**Table 3:** *ACE* haplotype frequencies in hypertensive and non-hypertensive individuals.

Haplotypes	Block 1	HT	NHT	OR (95% CI)	*P* value
**H1**	***A-A-G-C-A***	**0.489**	**0.556**	**0.77 (0.66**–**0.97)**	**0.023**
**H2**	***G-G-A-T-G***	**0.426**	**0.346**	**1.41 (1.11**–**1.80)**	**0.004**
H3	*A-G-A-T-G*	0.011	0.022	NS	—
H4	*A-G-A-C-A*	0.013	0.014	NS	—

HT, hypertensive; NHT, non-hypertensive; OR, odds ratio; CI, confidence interval; NS, not significant. The order of the polymorphisms in the haplotypes is according to the positions in the chromosome (rs4335, rs4344, rs4353, rs4362, rs4363).

**Table 4:** Risk assessment according to haplotypes using the codominant inherence model.

Model	Haplotypes	OR	95% CI	P value
	H1/H1	1		
Co-dominant	H1/H2	2.00	1.294–3.118	0.002
	H2/H2	2.09	1.180–3.704	0.011

OR; odds ratio, CI; confidence interval.

Adjusted by age, gender, BMI, triglycerides, alcohol consumption, and smoking.

### Correlation between haplotypes and serum ACE levels

**Figure 2:**
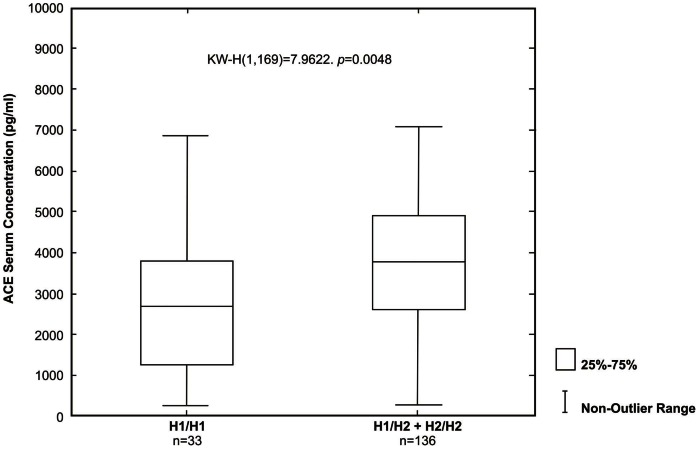
Graphic representation of serum ACE concentrations according to haplotypes. The limits of the boxes represent the middle 50% of the data values; the extent of the lines encompass the interquartile range with extreme outlying data points shown as such. The central line within each box represents the median. H1/H1 = homozygous for protection, H1/H2 = heterozygous protection/risk and H2/H2 = homozygous for risk. Serum ACE concentrations were measured in patients without ACE inhibitors treatment.

Serum ACE levels were analyzed in 33 hypertensive individuals homozygous for the H1 protective haplotype (H1/H1) and in 136 hypertensive individuals with at least one H2 risk haplotype (H1/H2 and H2/H2) (Fig. 2). Significant elevations in serum ACE concentrations were found in individuals with risk haplotypes (H2/H2 and H1/H2) (median 3802.37 pg/ml.) as compared with H1/H1 individuals (median 2692.73 pg/ml; *P* = 0.0048).

## Discussion

Essential hypertension involves interactions among genetic, environmental, demographic, vascular, and neuroendocrine factors. The etiologic role of genes implicated in the renin-angiotensin system (RAS) has been widely studied. Several clinical studies have reported an association between *ACE* polymorphisms and pathological phenotypes [19]–[28]. In the present study, we genotyped nine *ACE* gene polymorphisms in a group of Mexican individuals with and without essential hypertension. Six out of nine polymorphisms (rs4291, rs4335, rs4344, rs4353, rs4362 and rs4363) were associated with hypertension in our group of patients. These associations were independent from other hypertension-associated risk factors. The rs4343 polymorphism was not associated with hypertension perhaps because this polymorphism was not in HWE in the non-hypertensive individuals. Of the studied polymorphisms, only the rs4291 has been previously studied in patients with hypertension. Zhu et al. [29] reported that individuals with the rs4291 *T* allele showed increased blood pressure, in agreement with our observation in the Mexican population. On the other hand, the bioinformatics tools we used predicted that only the rs4335, rs4353 and rs4363 polymorphisms have a potential functional effect, and this variant is predicted to be in a binding site for the AML-1, GATA-1, and Srp40 transcription factors. The presence of specific alleles in these polymorphisms could result in loss (or reduction) of DNA binding of these transcriptional factors with important consequences in the expression of the enzyme. According to linkage disequilibrium analysis, five out of nine polymorphisms studied (rs4335, rs4344, rs4353, rs4362, rs4363) were in high linkage disequilibrium and four haplotypes were constructed, one of them associated with risk (H2: *GGATG*) and another with protection (H1: *AAGCA*) for hypertension. Zhu et al. [29] studied 10 polymorphisms in hypertensive patients from African-American and European-American populations. Our study included 8 of these 10 polymorphisms. In the study by Zhu et al. [29], a haplotype with three polymorphisms was associated with hypertension; however, the haplotype associated was different in African-American and European-American patients. In the African-Americans, the haplotype associated included the rs4343, rs4353, and rs4363 polymorphisms (*AAA* haplotype), whereas in European-Americans the haplotype included the rs4335, rs4343, and rs4344 polymorphisms (*GGG* haplotype). The risk haplotype in the Mexican Mestizo population includes the rs4335 *G* and rs4344 *G* alleles detected in European-American population and the rs4353 *A* allele detected in African-American individuals.

We also measured ACE serum levels to seek whether any haplotype was associated with this trait in hypertensive individuals. A limitation of our study is the fact that ACE levels were not measured in non-hypertensive individuals. Significant elevations in serum ACE concentrations were found in hypertensive individuals with the risk haplotypes when compared to individuals with the protective haplotype. This result supports the role of these haplotypes in the genetic susceptibility to developing essential hypertension. In a previous study, the relationship between the ACE levels and seven polymorphisms of the *ACE* gene was analyzed [30]. None of the polymorphisms studied by Zhu et al. [30] was included in our study. Zhu et al. [30] detected a haplotype associated with ACE levels. We suggest that these studies be extended to other ethnic groups to establish other regulatory regions in the *ACE* gene.

The most studied *ACE* gene polymorphism in this case is the insertion/deletion. In the study by Zhu et al. [12], two polymorphisms accounted for the variation of ACE concentration, *A2350G* had the most significant effect, accounting for 19% of the total variation in ACE, while rs4291 located in the 5′ section of the gene accounted for 6% of the variation. The effects of both these polymorphisms fitted best an additive model. After adjustment for the effect of *A2350G*, the *I/D* polymorphism was no longer associated with ACE, indicating that it is in LD with *A2350G* and unlikely to be a functional polymorphism. In this study, was established that the rs4291 *A* allele is associated with increased levels of ACE. This polymorphism was not associated with either hypertension or ACE levels in the Mexican Mestizo population. The association of other *ACE* polymorphisms with ACE levels is contradictory with positive and negative associations [31]–[34].

## Conclusions

In summary, our results suggest that the rs4291, rs4335, rs4344, rs4353, rs4362, and rs4363 *ACE* gene polymorphisms might play an important role in the development of hypertension in the Mexican population. In our study, it was possible to identify a risk haplotype (H2: *GGATG*) and a protective haplotype (H1: *AAGCA*) for hypertension. Individuals with the risk haplotype showed increased ACE plasma levels as compared to individuals with the protective haplotype.

## Supporting Information

Figure S1 **Haploview linkage disequilibrium graph of nine** ***ACE*** **gene polymorphisms.** Pairwise linkage disequilibrium coefficients D×100 are shown in each cell (D ´ values of 1.0 are not shown). Standard color scheme of Haploview was applied for linkage disequilibrium color display (logarithm of odds [LOD] score ≥2 and D  = 1, shown in bright red; LOD score ≥2 and D ´**<**1 shown in shades of pink/red; LOD score ≤2 and D ´**<**1 shown in white). D values of 1.0 are not shown. Numbers in boxes and parentheses represent D values and r2 values after the decimal point, respectively.
(TIFF)

Table S1 **Genetic Polymorphisms studied in the** ***ACE*** **gene.**
(DOC)
