# Supplementary figures and images for: Single Nucleotide Polymorphisms of the Angiotensin-Converting Enzyme (ACE) Gene Are Associated with Essential Hypertension and Increased ACE Enzyme Levels in Mexican Individuals
Source: PLoS One. 2013 May 31;8(5):e65700. doi: 10.1371/journal.pone.0065700 (PMC3669228; doi:10.1371/journal.pone.0065700)

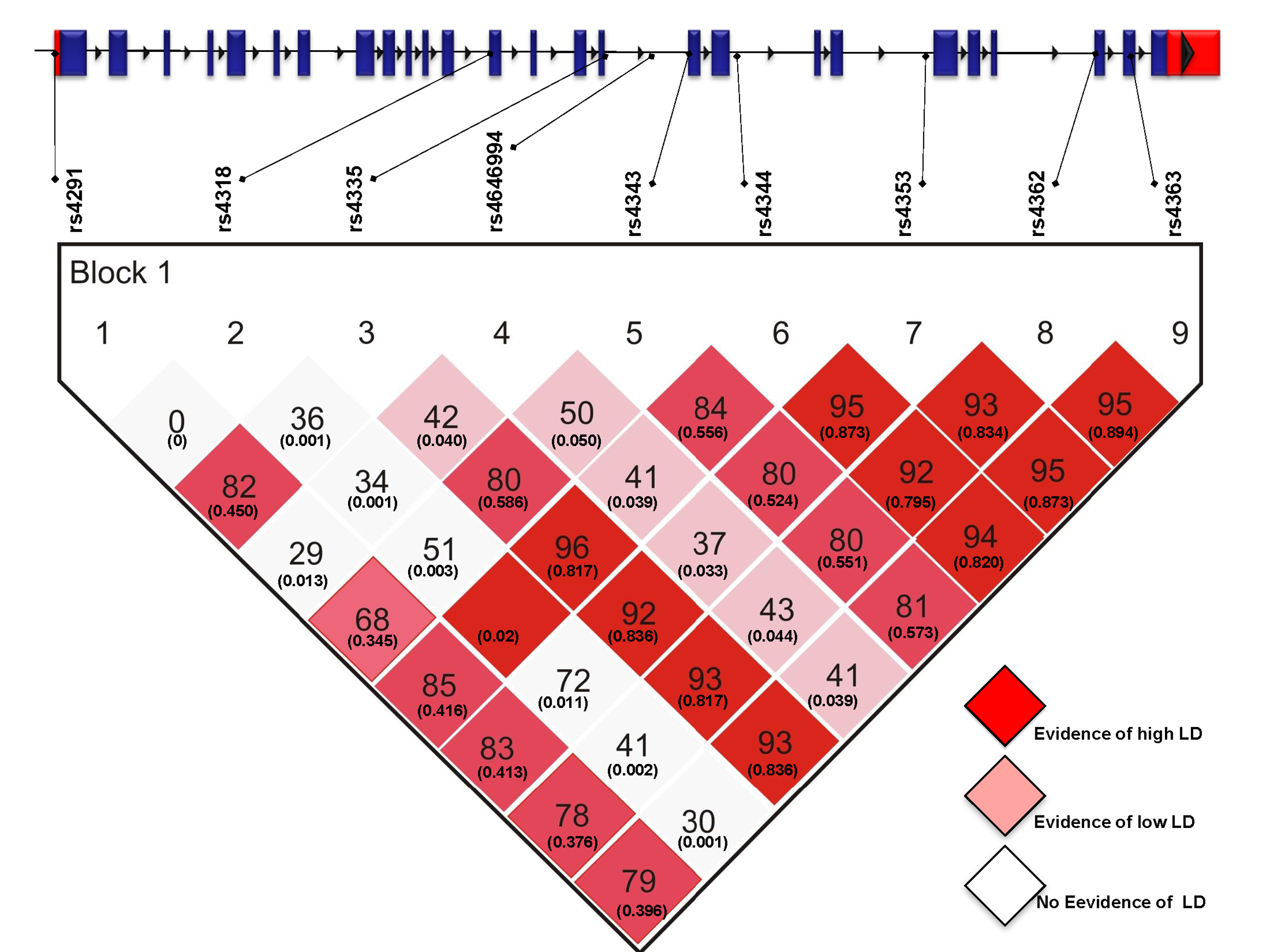

Supplement: Figure S1 — Haploview linkage disequilibrium graph of nine ACE gene polymorphisms. Pairwise linkage disequilibrium coefficients D×100 are shown in each cell (D ´ values of 1.0 are not shown). Standard color scheme of Haploview was applied for linkage disequilibrium color display (logarithm of odds [LOD] score ≥2 and D = 1, shown in bright red; LOD score ≥2 and D ´<1 shown in shades of pink/red; LOD score ≤2 and D ´<1 shown in white). D values of 1.0 are not shown. Numbers in boxes and parentheses represent D values and r2 values after the decimal point, respectively. (TIFF) [file pone.0065700.s001.tifF]
