# Supplementary material for: Single Nucleotide Polymorphisms of the Angiotensin-Converting Enzyme (ACE) Gene Are Associated with Essential Hypertension and Increased ACE Enzyme Levels in Mexican Individuals
Source: PLoS One. 2013 May 31;8(5):e65700. doi: 10.1371/journal.pone.0065700 (PMC3669228; doi:10.1371/journal.pone.0065700)
Supplement: Table S1 — Genetic Polymorphisms studied in the ACE gene. (DOC) [file pone.0065700.s002.doc]

**Supplementary material**

**Table S1. *ACE*** gene polymorphisms tested

| No.^a^ | Polymorphism^b^ (minor allele) | dbSNP^c^ | Chr position (pb) | Location in gene |
| --- | --- | --- | --- | --- |
| 1. | *A-239T (T)* | rs4291 | 61554194 | 5´near region |
| 2. | *A7941G (G)* | rs4318 | 61562373 | Exon 13 |
| 3. | *A10539G (A)* | rs4335 | 61565025 | Intron 16 |
| 4. | *I/D[(287BP ALU)/-] (I)* | rs4646994 | 61565904:61565905 | Intron 16 |
| 5. | *A11599G (A)* | rs4343 | 61566031 | Exon 17 |
| 6. | *A12292G (A)* | rs4344 | 61566724 | Intron 18 |
| 7. | *A15990G (G)* | rs4353 | 61570422 | Intron 20 |
| 8. | *C19329T (C)* | rs4362 | 61573761 | Exon 25 |
| 9. | *A20060G (A)* | rs4363 | 61574492 | Intron 24 |

^a^Order of the polymorphisms is according to the chromosomal positions

^b^Given Name according to NCBI.

^c^SNP ID in database dbSNP.
